# Frailty and intrinsic capacity: integrating complementary concepts to promote healthy ageing and transformation of care

**DOI:** 10.1093/ageing/afag138

**Published:** 2026-05-21

**Authors:** Matteo Cesari, Marco Canevelli, Jotheeswaran Amuthavalli Thiyagarajan, Hidenori Arai, Praset Assantachai, Piu Chan, Jagadish K Chhetri, Eduardo Ferriolli, Leon Geffen, Celia L Gregson, Giovanni Guaraldi, Luis Miguel Gutierrez Robledo, Sonia Hammami, Hyobum Jang, Sebastiana Kalula, Arvind Mathur, Reshma Aziz Merchant, Déborah Oliveira, Mônica R Perracini, Mirko Petrovic, Maria Cristina Polidori-Nelles, Leocadio Rodriguez Mañas, John W Rowe, Saniya Sabzwari, Ritu Sadana, Yuka Sumi, Olena Tomarevska, Bruno Vellas, Renuka Visvanathan, Jean Woo, Finbarr C Martin

**Affiliations:** Ageing and Health Unit, Department of Maternal, Child, Newborn, Adolescent Health and Ageing, World Health Organization, Avenue Appia 20, 1211 Geneva 27, Switzerland; Department of Human Neuroscience, Sapienza University, Rome, Lazio 0185, Italy; Ageing and Health Unit, Department of Maternal, Child, Newborn, Adolescent Health and Ageing, World Health Organization, Avenue Appia 20, 1211 Geneva 27, Switzerland; National Center for Geriatrics and Gerontology, 7-430 Morioka-cho, Obu, Aichi 484-0038, Japan; Faculty of Medicine, Mahidol University and Siriraj Hospital, Bangkok City 10700, Thailand; National Clinical Research Center for Geriatric Disorders: Xuanwu Hospital, Capital Medical University, 45 Changchun Street, Xicheng District, Beijing 100053,Beijing, China; National Clinical Research Center for Geriatric Disorders: Xuanwu Hospital, Capital Medical University, 45 Changchun Street, Xicheng District, Beijing 100053,Beijing, China; Nepalese Society of Gerontology and Geriatrics, Bhaktapur, Ward 3, Bhaktapur, Nepal; Division of Geriatrics, Department of Internal Medicine, University of São Paulo Medical School, Avenida Professor Luciano Gualberto, 908, São Paulo, São Paulo, SP, Brazil; The Albertina and Walter Sisulu Institute of Ageing in Africa, Department of Medicine, University of Cape Town, Western Cape, 7935, Cape Town, South Africa; Global Health and Ageing Research Unit, University of Bristol, Level 1 Learning and Research Building, Southmead Hospital, BS10 5NB, Bristol, UK; The Health Research Unit Zimbabwe, Biomedical Research and Training Institute, 10 Seagrave Avondale, Harare, Zimbabwe; Department of Surgical, Medical, Dental, and Morphological Sciences, University of Modena and Reggio Emilia, Via Università 4 - 41121, Modena, Emilia-Romagna, Italy; Instituto Nacional de Geriatría, Av. Contreras 428 Col. San Jerónimo Lídice 10200, Mexico City, Mexico; Department of Internal Medicine and Research Laboratory LR 12ES05, University of Monastir, Avenue Taher Hadded B.P. 56, Monastir, Tunisia; Ageing and Health Unit, Department of Maternal, Child, Newborn, Adolescent Health and Ageing, World Health Organization, Avenue Appia 20, 1211 Geneva 27, Switzerland; The Albertina and Walter Sisulu Institute of Ageing in Africa, Department of Medicine, University of Cape Town, Western Cape, 7935, Cape Town, South Africa; Asian Centre for Medical Education Research & Innovation, SN Medical College, Shastri Nagar, Residency Road, Jodhpur, Rajasthan 342001, India; Department of Medicine, National University Hospital, 21 Lower Kent Ridge Road Singapore 119077, Singapore; Faculty of Nursing, Universidad Andrés Bello, 121,Avenida Bellavista 8420507 Santiago Metropolitana de Santiago, Santiago, Chile; Millenium Institute for Care Research (MICARE), Avenue Libertador Bernardo O´Higgins 340, Casa Central, Santiago, Chile; Master’s and Doctoral Program in Physical Therapy, Universidade Cidade de São Paulo, Rua Cesário Galeno, 448/475, Tatuapé, Sao Paulo, SP, Brazil; Section of Geriatrics; Department of Internal Medicine and Paediatrics, Ghent University, Gent 9000, Belgium; Ageing Clinical Research, Department II of Internal Medicine and Center for Molecular Medicine, University Hospital Cologne, Kerpener Str 62 · 50937 Cologne,, Germany; Cologne Excellence Cluster on Aging and Aging-Associated Diseases (CECAD), University of Cologne, Cologne, Germany; Servicio de Geriatría, Hospital Universitario de Getafe, Calle Toledo 12, 28901, Getafe (Comunidad de Madrid), Madrid 28905, Spain; The Columbia Aging Center and the Department of Health Policy and Management Mailman, School of Public Health, Columbia University, , 722 W 168th St, New York, NY 10032, USA; Department of Family Medicine, The Aga Khan University, Stadium Road, P.O. Box 3500, Karachi 74800, Sindh, Pakistan; Ageing and Health Unit, Department of Maternal, Child, Newborn, Adolescent Health and Ageing, World Health Organization, Avenue Appia 20, 1211 Geneva 27, Switzerland; Ageing and Health Unit, Department of Maternal, Child, Newborn, Adolescent Health and Ageing, World Health Organization, Avenue Appia 20, 1211 Geneva 27, Switzerland; D.F. Chebotarev Institute of Gerontology of the National Academy of the Medical Sciences of Ukraine Kyiv, vulytsia Volodymyrska, 54 Kyiv City, Ukraine; IHU HealthAge, Gérontopôle de Toulouse, Centre Hospitalo-Universitaire de Toulouse, 2 Rue Viguerie, 31300, Toulouse, France; CERPOP, UPS/Inserm 1295, 37 Allées Jules Guesde, 31400 Toulouse, France; Adelaide Geriatrics Training and Research with Aged Care (GTRAC) Centre; School of Medicine, College of Health, Adelaide University, 4 North Terrace, Adelaide South Australia 5000, Australia; Basil Hetzel Institute, Central Adelaide Local Health Network, 37A Woodville Rd, Adelaide South Australia 5011, Australia; Jockey Club Institute of Ageing, Suite 602, 6/F, Yasumoto International Academic Park, The Chinese University of Hong Kong, Shatin, New Territories, Hong Kong, China; Faculty of Life Sciences & Medicine, Population Health Sciences, King’s College London, Great Maze Pond, London SE1 9RT, UK

**Keywords:** geriatrics, public health, ageing, care, older people

## Abstract

As the global population ages, especially in low- and middle-income countries, there is an urgent need to rethink how health in older age is understood and addressed. Frailty has long served as a clinical construct to identify vulnerability and guide tailored, specialist care for older people. In 2015, the World Health Organisation introduced the concept of intrinsic capacity (IC) as part of its healthy ageing framework, offering a structured, capacity-based approach to promote functional ability. While conceptually distinct, frailty and IC are complementary. Frailty highlights the need for specialised care in complex cases, whereas IC supports early intervention and prevention across broader populations. This paper explores their differences, areas of overlap and how their integration can support a continuum of care that spans primary to specialist settings. Integrating these concepts connects prevention, health promotion and complex care management. By aligning clinical and public health perspectives, the combined use of frailty and IC offers a holistic, person-centred approach to care system transformation, with the potential to drive coordinated strategies that strengthen both geriatric practice and public health across diverse populations.

## Key Points

- Frailty helps identify when an older person is highly vulnerable and needs more specialised care.
- Intrinsic capacity describes the physical and mental abilities a person can use to maintain their functional ability.
- Using frailty together with intrinsic capacity links early prevention with support for complex needs across the care continuum.

## Global ageing—more than a challenge of numbers

**Figure 1:**
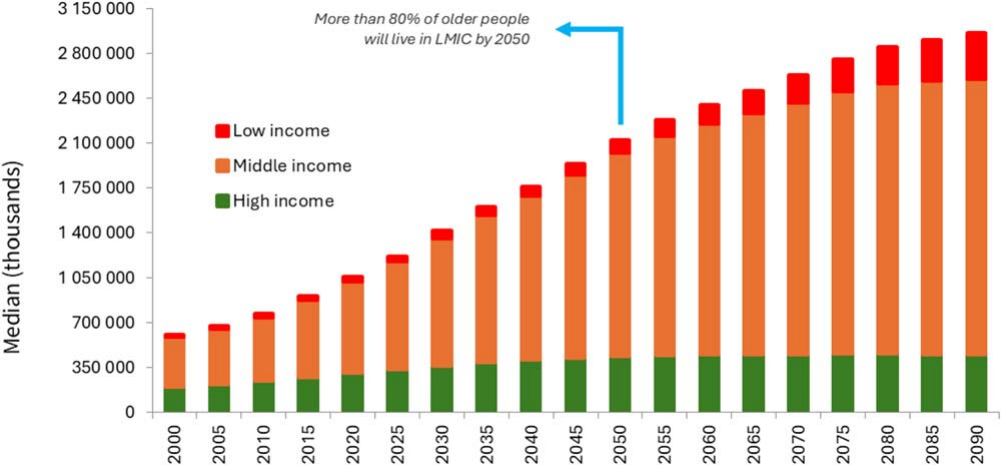
Number of persons aged 60 years and above over time, by country income. Data are from the *World Population Prospects 2024* (https://population.un.org/wpp/).

The world is ageing at an unprecedented pace, with most of the demographic shift occurring in regions with few high-income countries (HICs). By 2050, over 80% of people aged 60 and above will live in low- and middle-income countries (LMICs; Figure 1) [1], where ageing is accelerating amid limited health system capacity [2]. While HICs must adapt well-established systems to older populations, LMICs face a dual challenge: upscaling to universal health coverage and building care models suited to rapidly changing needs.

A shift in health trajectories accompanies this demographic change [3]. Instead of premature, and often sudden deaths, more people now live for years with fluctuating and unstable health in older age [4]. This reflects the cumulative impact of stochastic biological processes that drive ageing [5], marked by a gradual reduction of the organism’s physiological reserves and greater vulnerability to internal and external stressors [6, 7]. Consequently, age-related health conditions often appear in complex, overlapping ways that challenge traditional diagnostic categories. These patterns question conventional care models, which remain mainly disease-focused and poorly equipped to meet the multidimensional needs of older adults [8], especially in settings where healthcare systems are under-resourced or fragmented [9].

In HICs, the challenge lies in age-attuning primary care and organ-specialty dominated secondary care systems. In LMICs, the task is to reorient and expand systems responsive to the realities of ageing. The United Nations Decade of Healthy Ageing (2021–2030) [10] underscores the need for system-wide transformation rather than piecemeal adaptation.

Geriatric medicine has been central to this shift, particularly through the concept of frailty, which captures the age-related decline in physiological reserves and associated clinical risks [11–14]. Frailty shifts the way illness manifests in older adults (i.e. often with non-specific symptoms, functional decline and social complexities), highlighting the need for comprehensive, person-centred, multidisciplinary care.

At the population level, the World Health Organisation (WHO)’s healthy ageing framework has advanced the concept of intrinsic capacity (IC, the composite of an individual’s physical and mental capacities) as a complementary perspective [15]. Preserving IC, alongside supportive environments, strengthens functional ability and reframes care from disease management to resilience-building.

### Complementarity of frailty and IC

Although frailty and IC differ in their origins, methods and applications [16–19], they share a transformative goal: to move beyond chronological age and static diagnostic categories towards embracing a dynamic understanding of ageing [16, 20, 21]. Both concepts have conceptual validity and practical usefulness; they are not competing but have emerged to address different clinical and policy needs in the context of ageing. Frailty, rooted in geriatric medicine and increasingly being applied in organ-based specialty services, highlights the vulnerability linked to significant loss of age-related physiological reserves and the increased risk of adverse outcomes, and therefore, the need for tailored clinical responses. IC, developed within a public health lifecourse framework, emphasises maintaining physical and mental capacities that support functional ability in older age, stressing the importance of preventive strategies.

Together, these constructs provide a more nuanced understanding of ageing, moving beyond fixed diagnostic categories to embrace a dynamic, multidimensional view of health, one that combines clinical insight with public health perspectives. This is especially relevant for clinicians and researchers deeply involved in frailty-related work, who may perceive IC as redundant or confusing. Rather than replacing frailty, IC provides a complementary framing aligned with preventive, longitudinal and person-centred approaches.

We propose that both frailty and IC can and should be incorporated into a unified framework for the care of older people. Each construct must respond to the underlying biological mechanisms of ageing and be guided by emerging insights from geroscience [22]. This alignment reflects two related continua: a biological continuum [23], in which shared ageing mechanisms shape both IC decline and frailty development; and a clinical continuum [24], where sequential or phased assessments offer complementary and sometimes distinct information, supporting early identification, risk stratification and interventions.

This integrated approach could support the development of person-centred care systems capable of addressing the complex and changing needs of older populations. To support this proposition, the article will examine in greater detail the concepts and operationalisations of frailty and IC, clarifying what unites and differentiates them, and how each contributes to a common understanding of high-quality, integrated care for older people across various health system contexts.

**Table 1:** Intrinsic capacity and the International Classification of Functioning, Disability and Health framework

	IC	ICF
*Definition*	All the physical and mental capacities that a person can draw on	A framework of health and health-related domains used to describe functioning and disability
*What it describes*	What a person can do	How a person functions in the daily life
*Focus*	Physical and mental capacities of the individual (i.e. cognitive, locomotor, vitality, vision, hearing and psychological domains)	Body functions and structures, activities, participation and contextual factors
*Role of environment*	Considered separately from IC	Integral component
*Use*	Used in the context of healthy ageing and to support preventive strategies	Commonly used in rehabilitation, disability and broader assessment of functioning

### The concept of frailty

Frailty originated as a concept within geriatric medicine in HICs, where clinical systems are predominantly shaped by disease-centric paradigms. It was introduced to capture the biological, clinical and social complexities of older individuals, providing a means to move beyond merely considering chronological age and disease-based methods. Frailty enables the proactive identification of vulnerability before the onset of the disabling cascade [25–27]. Although the term ‘frailty’ has long existed [28], its consistent application in clinical literature only gained traction in the early 2000s with the development of operational definitions.

Two influential models helped formalise the concept. The Frailty Index, developed by Rockwood and Mitnitski, quantifies the age-related accumulation of health deficits [6], and serves as a proxy for biological age [12]. In contrast, the phenotypic model proposed by Fried and colleagues focuses on a core of physical manifestations (i.e. slowness, exhaustion, weakness, sedentariness, involuntary weight loss), suggesting the existence of underlying biological mechanisms [29].

Despite their different emphases, both models converge on the central notion of vulnerability [30, 31], which defines a reduced capacity to respond to stressors, thereby increasing the risk of adverse outcomes (e.g. dependency, hospitalisations, institutionalisation and death). This shared focus has helped identify a target population requiring adapted care pathways, most notably through Comprehensive Geriatric Assessment (CGA; ‘a multi-dimensional, multi-disciplinary diagnostic and therapeutic process conducted to determine the medical, mental, and functional problems of older people with frailty so that a coordinated and integrated plan for treatment and follow-up can be developed’) [32, 33]. The principles behind frailty management were anticipated by earlier works on CGA, particularly those of Rubenstein and colleagues [34], who emphasised holistic evaluation and individualised care. Frailty is now recognised as a key factor in the management of communicable and noncommunicable diseases (e.g. human immunodeficiency virus (HIV) [35], cancer [36], cardiovascular disease [37], chronic kidney disease [38], chronic lung disease [39] or fractures [40]), underscoring the importance of personalised, integrated care for older people.

However, CGA remains resource-intensive and requires specialised skills, which are often lacking [41–43], especially in LMICs where population ageing is accelerating and geriatric expertise is urgently needed but limited. Furthermore, in higher-income settings where primary care has a prominent place in the ongoing health care and health promotion of older people, CGA has not been widely adopted as a discrete intervention within the continuum of care [44].

To address this challenge and promote preventive strategies for age-related conditions, it is essential to strengthen and develop capacity in primary care. Indeed, primary care plays a central role in delivering preventive interventions and is often the first point of contact for individuals from diverse social and economic backgrounds. Its broad reach and fundamental position within health systems make it an essential component in efforts to advance Universal Health Coverage and achieve the Sustainable Development Goals [45]. To promote high-quality care (including long-term care and support) close to where people live and age (i.e. ‘ageing in place’), basic skills for engaging with older adults, understanding their needs and priorities, delivering personalised interventions and ensuring the continuum of integrated care are increasingly needed outside specialist settings [46, 47]. In other words, the concept of age-attuned care, which recognises clinical vulnerability, should be adapted and decentralised from specialist settings to primary care, an effort that, to date, has only been attempted sporadically with mixed results [44]. Nevertheless, various care services aimed at managing older persons with frailty have been introduced over the years, sometimes gaining national recognition and adoption [48–50]. Understanding why such decentralisation is needed requires considering the nature of frailty itself.

Current evidence shows that frailty is dynamic and potentially reversible, with trajectories that include stability, improvement and deterioration. Frailty assessments increasingly support shared decision-making, advance care planning and care intensification [51]. This reflects the original intent of the frailty construct: (i) to identify highly vulnerable individuals whose clinical needs were often missed in routine disease-focused examinations and (ii) to enable tailored, integrated care pathways that address the full spectrum of their needs. However, in some research and clinical contexts, the holistic intent of frailty has been diluted by the proliferation of subtypes (e.g. cognitive frailty, liver frailty, social frailty, oral frailty) [52, 53], which fragment the concept into single-domain approaches. Re-affirming frailty as a multidimensional construct is essential to preserve its meaning and maintain alignment with the foundational principles of CGA.

### The concept of intrinsic capacity

The WHO’s ‘World Report on Ageing and Health’ [15] defines healthy ageing as ‘the process of developing and maintaining the functional ability that enables well-being in older age.’ This definition was later incorporated into the WHO’s ‘Global Strategy on Ageing and Health’ [54], forming the foundation of a conceptual framework built around three interrelated components: IC, environment (i.e. all the factors in the extrinsic world that form the context of an individual’s life) and functional ability (i.e. the health-related attributes that enable people to be and to do what they have reason to value).

Intrinsic capacity derives conceptually from the ‘International Classification of Functioning, Disability and Health (ICF)’ [55]. ICF provides a comprehensive framework for describing functioning and disability, adopting a bio-psycho-social model. IC focuses specifically on the individual’s body structure, functioning and activity capacity components, amalgamated as intrinsic physical and mental capacities (Table 1).

Keeping this conceptual separation is crucial: social determinants of health strongly influence the trajectories of IC, but IC itself is defined independently of these external influences. The ICF framework helps differentiate between the drivers of health, the health state and its functional consequences. This perspective challenges the notion of ageing as an unavoidable decline, instead viewing it as a dynamic process in which well-being can be supported through targeted actions at individual and/or environmental levels.

To operationalise IC, WHO conducted an evidence-based analysis using the ICF framework to identify domains essential for independent living in older age [56]. These domains include locomotor capacity, cognitive capacity, sensory capacity (later split into hearing and vision), psychological capacity and vitality (which encompasses the metabolic capacity of the person, decline being often captured phenotypically by undernourishment). These domains are clinically relevant, as impairments in IC often precede and contribute to the disabling cascade. For this reason, the IC construct is operationalised to include a broad spectrum of domains amenable to direct interventions. This is distinct from the approach taken in models of frailty [57]. Unlike some frailty models that encompass social or environmental factors [58], IC is intentionally defined as distinct from these influences. This separation enables a more precise assessment of the individual’s capacities, grounded in their biological foundation.

WHO has developed guidelines on the management of declines in IC, particularly in community and primary care settings [59]. Building on these guidelines, additional guidance was later introduced to support health and care workers in primary care in designing and delivering person-centred interventions that target impairments of IC in older adults [47].

Pilot studies demonstrate that IC can be feasibly operationalised in diverse care contexts [60]. However, several methodological challenges remain. The flexibility of tools, while necessary for global applicability, reduces comparability across populations and health systems. Clinically meaningful thresholds for change in each IC domain are still unclear, and repeated assessments may be difficult to scale. Furthermore, although IC is conceptually separate from environmental factors, the distinction can blur in practice (e.g. psychological capacity influenced by social isolation). Acknowledging these uncertainties is critical for interpreting evolving IC evidence.

### Combatting ageism

A growing concern about the concept of frailty is the stigma associated with the term. Although the operationalisation of frailty was developed to capture the complexity of older individuals and enable early identification of reversible conditions or mitigation of potential adverse treatment effects, it is often misused as a fixed prognostic label, which sometimes results in limiting treatment in non-geriatric specialist settings. Such misapplication reinforces ageist attitudes, suggesting frailty is irreversible and confers an obstacle to benefitting from (or even meriting) care. Even well-intentioned models risk ageist interpretations if not carefully implemented and communicated.

Qualitative studies consistently show that older individuals perceive ‘frailty’ as a negative and disempowering label, often equated with helplessness, dependency or terminal decline [61–67]. This perception can discourage engagement with care services and contribute to self-ageism. What was intended as a tool for inclusion and tailored care has, in some cases, been reframed in ways that exclude individuals from essential interventions.

In contrast, the WHO’s framework based on IC offers a more positive and empowering narrative. IC focuses on the capacities that individuals retain and can build upon, shifting the discussion from what is lost to what can be preserved, restored or enhanced. By focusing on what matters to the individual and what can be improved, the language of care becomes more hopeful, collaborative and motivating. It encourages older people to engage in their own care and supports practitioners in delivering interventions that are not only clinically appropriate but also respectful and enabling.

Importantly, IC maintains face validity, resonating with both older people and practitioners without the negative connotations often associated with ‘frailty.’ The terminology is generally perceived as clear and more acceptable [68]. However, IC is not immune to misuse: like frailty, it can be interpreted in ageist ways if reduced to a score or used to gatekeep access to care. The challenge lies less in the terminology than in its implementation and communication. Future research should explore how best to frame and communicate both concepts to minimise stigma while retaining clinical precision.

### Measuring the health of communities

Given its structured, multidomain nature, IC offers a practical framework for a consistent and meaningful assessment of older people across different levels of the health system. This approach may contribute to improved monitoring of population health trends and inform discussions about healthcare system performance, particularly in the context of ageing [69]. While further validation is needed, early studies suggest IC is associated with a range of health outcomes [70–74], underscoring its potential utility in service planning and system performance monitoring.

Beyond its conceptual value, IC holds practical significance for healthcare delivery. It can assist in identifying population groups or geographic areas in need of targeted support, fostering more responsive and equitable care. Its clarity and flexibility also make it suitable for use across diverse settings, including those with limited resources.

Importantly, WHO’s endorsement of IC provides a globally recognised and standardised framework, which is still lacking in the fragmented field of frailty. At the same time, the construct remains amenable to developments and modifications, at a minimum, to reflect emerging evidence. This, for example, has already been occurring with the domain of vitality, probably the most complex domain to capture [72, 75, 76].

### From capacity to complexity: linked, not alike

We propose that while frailty and IC are conceptually distinct, they are complementary (Table 2).

**Table 2:** Frailty and intrinsic capacity

Feature	Intrinsic capacity	Frailty
*Origin*	Public health framework (WHO)	Geriatric medicine
*Objective*	Promotion of healthy ageing and optimisation of functional ability	Delivery of person-tailored, adapted care
*Focus*	Physical and mental capacities	Deficits and vulnerabilities
*Population*	Middle-aged and older persons	Older persons with an enhanced risk of adverse health outcomes
*Assessment structure*	Standardised domains with flexible, contextualised tools	Multiple, heterogeneous models
*Stigma risk*	Generally lower, due to positive and capacity-focused framing	Often perceived as high, depending on context and communication
*Care model*	Integrated care for older people	Clinical, specialist-led CGA; specialistic component within an integrated care model
*Implementation*	Primary care and community; care settings with non-specialistic competencies in geriatric medicine	Specialist settings with competencies in geriatric medicine
*Policy implications*	Emphasis on prevention, community-based care, improved allocation and integration of resources in primary health care	Focus on risk stratification, resource allocation and specialised services
*Role in continuum of care*	Essential to guide early interventions and preserve functional ability	Critical for identifying those needing intensive or specialised care
*Equity*	Encourages inclusive, upstream strategies adaptable to diverse settings	May misleadingly be used to discriminate; may overlook early-stage needs, especially when adopted in settings without geriatric competencies

Abbreviations: CGA, Comprehensive Geriatric Assessment; WHO, World Health Organisation

**Figure 2:**
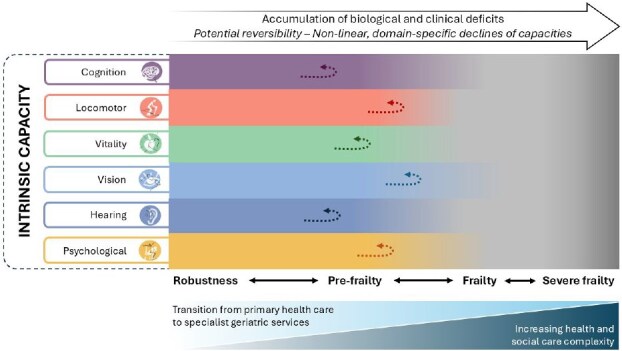
Conceptual transition from intrinsic capacity impairments toward frailty. The figure illustrates how accumulating deficits across IC domains can increase care complexity and lead to the need for specialist geriatric management. Declines in IC domains are heterogeneous, nonlinear and potentially reversible. Differential fading of domains is purely illustrative and does not imply a univocal order of decline. The horizontal scale (from robustness to severe frailty) represents a conceptual continuum that serves as a visual guide to increasing complexity. There is no fixed threshold for transition from primary health care to specialist geriatric care; this depends on contextual factors, such as available resources, capacity of care workers, system design and opportunities for intervention. The pathway is conceptual, not an empirically validated progression model.

By focusing on a structured set of physical and mental capacities that can be assessed with relatively simple tools, IC can extend a capacity-based approach among health and care workers, beyond those with specialised expertise in geriatric care, broadening access to assessment. While IC covers the entire spectrum of functioning, frailty marks the point at which clinical and social complexity may require specialist care. As depicted in Figure 2, declines in IC follow heterogeneous, domain-specific, nonlinear and potentially reversible trajectories. Progression toward frailty, by contrast, reflects the accumulation of biological and clinical deficits rather than a uniform or linear sequence.

At both the individual and population levels, the six domains of IC follow distinct trajectories, shaped by different determinants and associated with varied outcomes. Despite their differences, these domains interact closely over time, and the cumulative impact of multiple impairments may contribute to growing clinical complexity, eventually leading to the emergence of frailty. Given their heterogeneous, nonlinear impact on care, a CGA-based specialised approach becomes appropriate when significant declines occur. The threshold marking the onset of frailty should not be viewed solely as related to the biology of the individual. It also depends on the environment, specifically the availability of specialised care and the capacity of primary care to properly manage the needs and priorities of older people before referral.

### IC and frailty as part of the same framework

Positioning IC and frailty on the same continuum helps integrate two complementary geriatric frameworks serving different goals. IC supports a proactive, public health-oriented model of care, while frailty defines when specialist geriatric interventions are needed for more complex clinical situations.

The structured, domain-based approach of IC makes it adaptable across medical specialties and accessible to non-specialist providers, offering a shared language for interdisciplinary collaboration. Its use in disease-specific services (e.g. HIV care) illustrates how IC can guide personalised interventions beyond geriatric settings [77]. In these contexts, recognising an increased vulnerability alone is insufficient; interventions must aim to preserve IC and hence impact functional ability.

By organising interventions around specific domains of IC, health and care workers operating in disease-focused services are provided with intuitive, actionable entry points for personalised interventions. A key benefit of this model and its implementation also involves bridging the health and social sectors, exemplified by the active engagement of caregivers and community-based actors in the care plan, which enables interventions that are often overlooked (e.g. promoting self-management, mobilising social prescribing, adapting living environments and offering caregiver support).

However, IC cannot serve as a template to replace the CGA, which remains essential for managing frailty [33, 44]. IC focuses on specific capacities, and the care pathways for managing impairments in these domains must be tailored to their unique determinants. Differently, CGA is a clinical methodology designed to evaluate a broader set of areas not covered by IC (e.g. continence, pain, polypharmacy, fall and fracture risk, comorbidities and psychosocial engagement), many of which require specialised approaches.

Expanding the construct of IC to cover all domains typically addressed by CGA would risk diluting the original purpose to offer a consistent evidence-informed and capacity-oriented framework for primary care. Such expansion could also create pressure to incorporate condition-specific assessments (e.g. for hypertension, chronic kidney disease or chronic obstructive pulmonary disease), thus reducing the clarity and usefulness of the IC construct. However, it is important to recognise that the structured assessment of IC serves as an opportunistic entry point during routine contacts, helping identify impairments that may require further evaluation [47]. Its simplicity and face validity support feasibility even in largely reactive primary care systems. The Integrated Care of Older People (ICOPE) approach is highly adaptable and can be delivered by non-specialist health and care workers within a multidisciplinary team, ensuring efficiency and scalability. While it provides a simplified approach to person-centred care, it does not replace the depth and complexity of a CGA. Instead, it can trigger referral to specialists for a full CGA when appropriate. In this way, the IC construct remains conceptually distinct and protected, while informing the level and type of response needed.

Public health implementation illustrates how the ICOPE approach may benefit from existing geriatric resources and frailty infrastructures. In Mauritius, the national ICOPE strategy integrates the basic assessment conducted in primary care with geriatric-led training and specialist escalation for complex cases, reflecting established frailty and CGA referral mechanisms [78]. Similar alignment is seen in sub-Saharan Africa, where geriatric units in Cameroon and Senegal incorporate the ICOPE approach as an extension of geriatric and frailty-oriented services [79]. Comparable approaches also exist in Kuwait, where geriatricians support primary health care teams and serve as referral points within a system with limited specialist capacity [80]. For contrast, the experience from France shows how digital tools connect IC assessments (whether self-administered or conducted in primary care) to specialist geriatric management, demonstrating that the ICOPE approach can operate in a mature system without implying direct transferability to LMIC contexts [81].

### Considerations on the measurement of frailty and IC

The tools used to assess frailty and IC require careful consideration, as they can introduce ambiguity and create uncertainty. Translating theoretical constructs into practice involves both qualitative and quantitative assumptions (e.g. choosing variables and cut-off points), which may be arbitrary and context-dependent. This is especially relevant in LMICs, where sociocultural diversity and limited validation can affect the applicability of widely used instruments.

IC is not tied to specific tools. While some are advised for their accessibility, local adaptation is essential [47]. This flexibility helps avoid the methodological debates that have long challenged frailty research, where excessive focus on defining a ‘perfect’ tool has often overshadowed the clinical goal of identifying vulnerability [82]. A single ‘gold standard’ for frailty is unlikely, given its biological, clinical and contextual heterogeneity [83].

Although IC promotes a positive, capacity-based framing, many tools still capture deficits and are often used within CGA. This reflects the tension between public health aims and clinical application, but aligns with the overarching goal of delivering early, person-centred interventions.

## Conclusions

Frailty and IC offer complementary perspectives that strengthen geriatric and public health approaches to ageing. Frailty emphasises vulnerability and the need for specialised care, while IC provides a structured framework to support healthy ageing and guide interventions across broader populations. Integrating the two constructs enables a more holistic, person-centred lifecourse approach to care.

The domain-based structure of IC and its global relevance provide a practical tool for applying geriatric principles in daily practice, without replacing the need for CGA in more complex cases of older adults with frailty. Ultimately, operationalising this integration will require investment in training, workforce development and digital health tools to ensure scalability across diverse health systems.
